# Utilizing Soil Centrifugation for Accurate Estimates of Carbon Dioxide Removal via Enhanced Rock Weathering

**DOI:** 10.1021/acs.est.5c03699

**Published:** 2025-12-15

**Authors:** Gregory Jones, Ziyan Zhang, Katherine Clayton, Lena Lancastle, Athanasios Paschalis, Bonnie Waring

**Affiliations:** † Department of Life Sciences, 685422 Imperial College London, Silwood Park, Ascot SL5 7PY, U.K.; ‡ Department of Civil and Environmental Engineering, 120731 Imperial College London, London SW7 2AZ, U.K.; § School of Biosciences, University of Sheffield, Sheffield S10 2TN, U.K.; ∥ Department of Civil and Environmental Engineering, University of Cyprus, Nicosia 20537, Cyprus

**Keywords:** carbon dioxide removal, pore water, alkalinity, monitoring, enhanced weathering, land-based

## Abstract

Enhanced rock weathering (ERW) is a promising CO_2_ removal (CDR) strategy that aims to accelerate the natural process of silicate weathering to increase soil pore water alkalinity and sequester CO_2_. However, the measurement, reporting, and verification (MRV) of ERW remains challenging due to existing limitations of aqueous-phase sampling methodologies, such as passive and tension lysimeters, which may not fully capture weathering fluxes across varying soil moisture conditions. This study assesses the potential of a centrifugation-based pore water extraction method to improve the accuracy and reliability of ERW measurements. Using a forest ERW trial in Wales, UK, we compared the chemistry of soil pore waters obtained via lysimeters and centrifugation from feedstock-amended and control plots. The centrifugation method detected elevated total alkalinity and Ca concentrations in soil pore waters from feedstock-amended soils, whereas the effect of feedstock amendment was not detectable in pore waters extracted with lysimeters. The high tensions applied during centrifugation likely capture weathering products dissolved in meso- and micropore water, which lysimeters cannot extract. These findings suggest that centrifugation provides a scalable, low-cost approach for ERW monitoring, with implications for improving existing MRV protocols.

## Graphical abstract

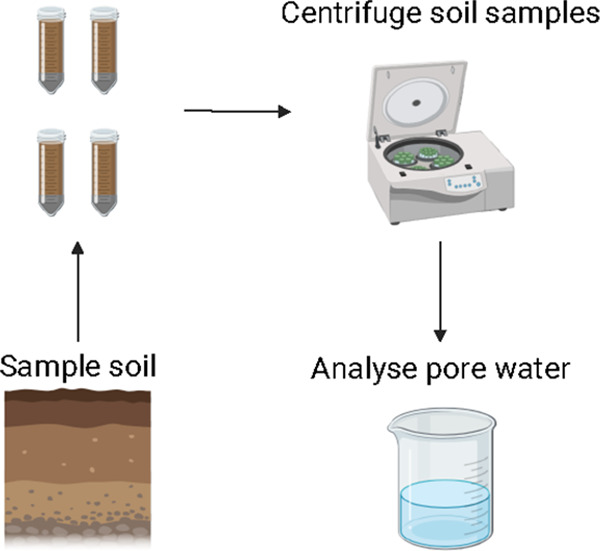

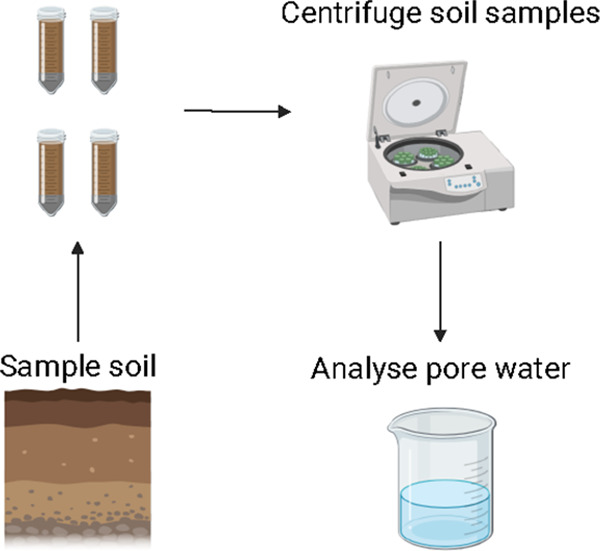

## 1 Introduction

Carbon dioxide removal (CDR) must be implemented to limit the effects of climate change.^1,2^ There is a need for CDR technologies to be both scalable and durable.^3^ One CDR method that shows such potential is enhanced rock weathering (ERW). ERW accelerates silicate weathering of Ca- and Mg-rich feedstocks to capture atmospheric CO_2_ through surface geochemical reactions within the soil column, producing base cations (e.g., Ca^2+^, Mg^2+^) and dissolved inorganic carbon (DIC, principally HCO_3_^–^). These weathering products are then transported through the soil profile and either precipitate as pedogenic carbonates or are eventually transported via ground or surface waters to the ocean, where they may be sequestered for thousands of years.^4–12^

Given the urgent need to implement CDR and the immaturity of technology-driven CDR strategies, such as direct air capture, a broad consensus has emerged advocating for the rapid upscaling of ERW.^4,5,13–19^ This will require new methods for accurate, low-cost, and scalable measurement, reporting and verification (MRV) of CO_2_ capture via ERW. However, it is currently challenging to assess the efficacy of ERW due to the lack of a universally agreed-upon method for in situ quantification of rock weathering.

Multiple methods are currently employed to estimate the extent of ERW-induced CDR at a given site, including solid- and aqueous-phase approaches.^18^ Aqueous-phase approaches typically involve collecting soil leachate or pore water with rhizon samplers or soil lysimeters (suction cup or tension) at set depths within the soil column.^20,21^ These enable in-field measurements of base cation release from weathering rock and indirect quantification of DIC via total alkalinity (TA) and pH.^22^ Moreover, anion concentration measurements (e.g., Cl^–^, NO_3_^–^, SO_4_^2–^) can determine whether weathering reactions are driven by carbonic acid (which results in the capture of CO_2_) or strong acids (which weather rock without directly sequestering any carbon).^4,21–23^ By contrast, solid-phase approaches determine reactant^24,25^ (e.g., Ca^2+^, Mg^2+^) losses from mineral phases^26,27^ and do not directly quantify inorganic carbon fluxes (although it is possible to measure changes in soil carbonates). These produce time-integrated weathering rate estimates but cannot provide information about the export of these products from weathering sites.^18^ Current MRV protocols for the ERW sector use both methods.^28^ Because each has specific advantages and limitations,^20^ integrating their benefits could provide a more holistic perspective on CDR via ERW.

This study focuses on advancing aqueous-phase methodologies to address current challenges in monitoring and quantifying ERW-induced CDR. Aqueous-phase measurements offer a relatively inexpensive method for estimating weathering fluxes and enable the identification of weathering hotspots, together with a better understanding of whether and how weathering products are exported from the feedstock application site. However, widely used lysimeters and rhizon samplers may not accurately capture the weathering flux from heterogeneous soil layers, as they typically extract water from a limited soil volume, which may not represent broader spatial variability.^29^ For example, passive lysimeters only collect gravitationally drained water, which may not capture the influence of finer pore-scale interactions in the soil matrix,^28–30^ where much of the chemical dissolution relevant to ERW occurs.^32,33^ Lysimeters and rhizon samplers also disturb soil structure, influencing natural flow paths and potentially affecting collected pore water composition.^34^ Such alterations may artificially perturb dissolution kinetics and weathering rates, impacting CDR estimates.

Moreover, the timing and frequency of lysimeter and rhizon sampling are limited to periods when the soil water content (SWC) is sufficiently high for pore water extraction, which may lead to temporal gaps in data (Figure 1). This can result in an incomplete understanding of weathering processes at the site of feedstock application and temporal fluctuations in CDR.^35,36^ Therefore, it is essential to develop an aqueous-phase method that captures the temporal variability of pore water chemistry and enables sample collection at SWC levels where lysimeters are typically ineffective (Figure 1).

**Figure 1:**
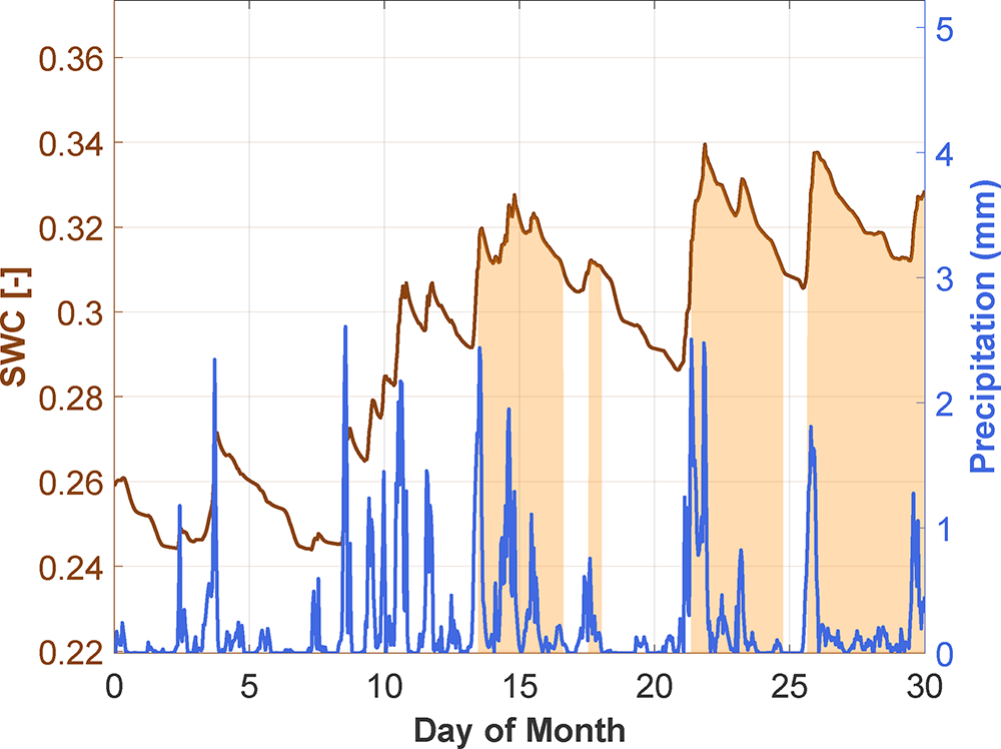
Sampling time points appropriate for lysimeter pore water extraction (orange shaded regions) in relation to soil water content (SWC) and corresponding daily precipitation. An SWC sampling threshold (SWC > 0.31) corresponds to an estimated water potential of approximately −30 kPa. Data was taken from ERA5-Land^41^ and corresponds to conditions at our field site in July 2023 (see Methods, Section 2.2 for further details).

Combining some of the benefits of solid-phase approaches, such as ease of field sampling^18^ with the advantages of aqueous sampling techniques, we propose a novel aqueous-phase extraction approach, the centrifugation pore water extraction sampling method, to obtain and analyze soil pore waters. Centrifugation extraction has long been employed to obtain interstitial water from rock and soil samples^31,37,38^ and could act as an accurate, low-cost method to determine terrestrial CDR via ERW. The central tenet of this approach involves centrifuging soil samples at high angular velocities to extract interstitial pore water. This water can then be examined following typical aqueous-phase analytical pathways (e.g., by examining total alkalinity (TA), base cation, and anion concentrations) to quantify CDR rates. In contrast to lysimeters, which apply between 0.04 and 0.1 MPa, and rhizon samplers, which apply approximately 0.05 MPa,^39^ centrifugation exerts pressures up to 1.5 MPa,^31^ exceeding the permanent wilting point of most soils.^38^ Therefore, centrifugation extraction may extract meso- and micropore water, revealing additional weathering products and insights into feedstock dissolution dynamics combined with more robust CDR estimates.

The objective of this study was to evaluate whether (1) the chemistry of the pore water derived via centrifugation extraction was comparable to that of lysimeters and (2) the soil centrifugation pore water extraction method provides a reliable method for sample extraction necessary for accurate CDR estimations through silicate feedstock weathering.

## 2 Methods

### 2.1 Site and Treatment Description

The experimental area was located in Cynghordy, south Wales (52°3′49″ N, 3°44′44′′ W, 219 m a.s.l.). The land was used as sheep pasture for at least 100 years before a reforestation experiment was established in 2019. The mean annual temperature was 9.6 °C, and the mean annual precipitation was 1566 mm (30-year climate averaging period, Met Office). The soil was classified as a well-draining fine loam, specifically a Chromic Mollic Endokeletal Umbrisol, within the MANOD (611c) soil association.^40,41^ The soil comprised of 36% sand, 24% clay, and 5.1% soil organic C.^42,43^

The study used a randomized block design (*n* = 4 blocks sampled for this experiment; Figure 2). Each block consisted of nine experimental plots, representing different treatment combinations of tree type (mixed native broadleaf or Sitka spruce), feedstock application (added or not), a microbial inoculation treatment (added or not), and a control treatment where no trees were planted and no feedstock was added.^44^ We only sampled plots from the “control” soil inoculation treatment for the measurements described in this paper. With 4 plots per block (factorial combination of feedstock addition and forest type) and 4 blocks, our final sample size was 16 plots per sampling time point. While the 16 plots varied in shape, the mean plot area was 1,598 m^2^ (range: 1,145 to 1,649 m^2^). Although treatments were applied across entire plots, samples were extracted from a central 20 × 20 m (0.04 ha) area. Trees were planted at a density of 2,500 trees ha^–1^ in May through June of 2021. Coniferous plots exclusively contained *Picea abies* (Sitka spruce), while broadleaf plots included *Sorbus aucuparia* (6%), *Prunus avium* (19%), *Populus tremula* (5%), *Betula pubsesns* (35%), *Alnus glutinosa* (22%), and *Quercus robur* (13%).

**Figure 2:**
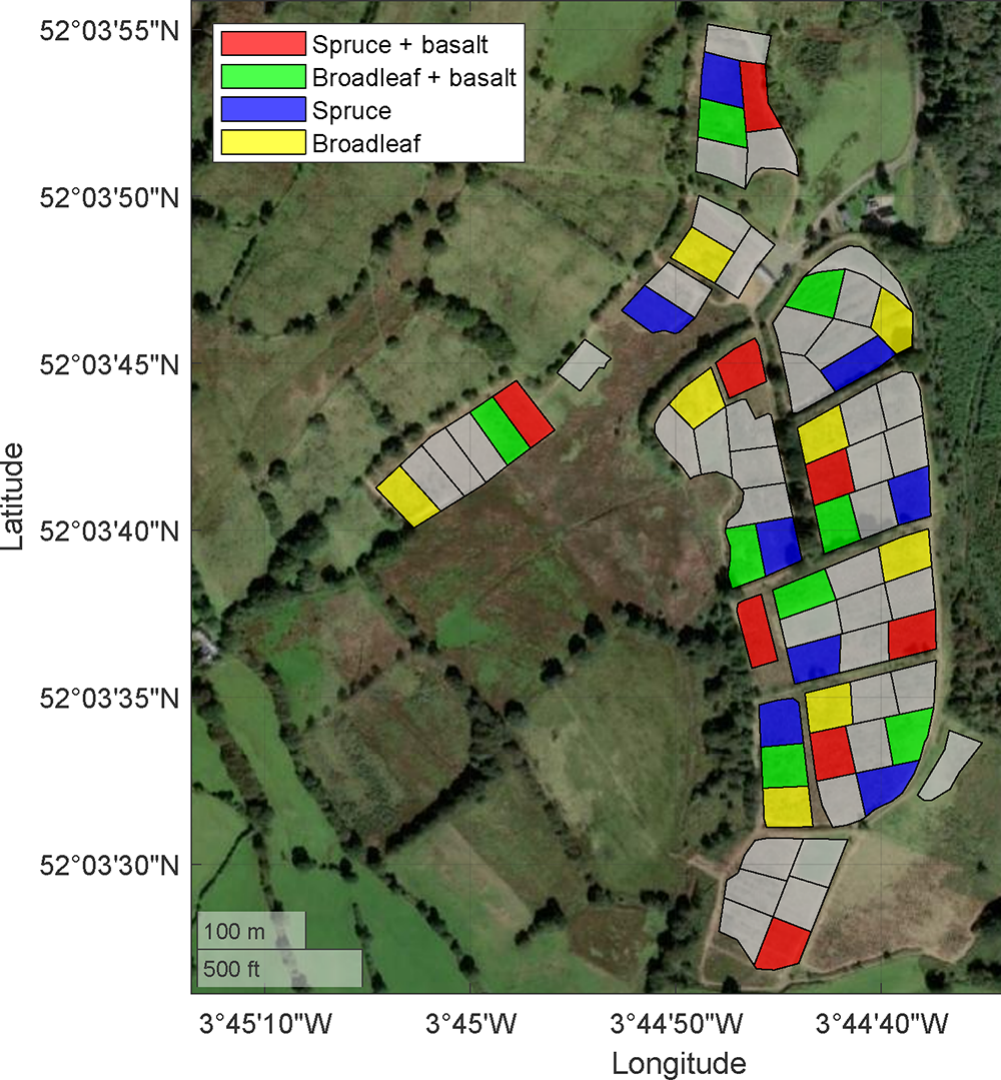
Experimental design of the Glandwr Forest study, displaying the arrangement of different treatment areas. Shaded regions denote plots present at the site but not sampled in this study.

A metabasalt from Builth Wells (Supplementary Section 1) was surface applied using a miniature bucket spreader in November 2020 (40 t ha^–1^, 4 mm mean particle size, median particle size 27 μm, Figures S1 and S2) and June 2023 (24 t ha^–1^, 2 mm mean particle size). Feedstock elemental composition was analyzed via X-ray fluorescence (XRF) spectroscopy (Zetium, Malvern Panalytical Ltd., UK) (*n* = 4).^44^ X-ray diffraction (XRD) analysis was performed on randomly oriented powder samples (*n* = 5) using a Rigaku miniflex diffractometer, with semiquantitative analysis via PANalytical Highscore Plus and ICDD PDF-4+ database (Table S1).

### 2.2 Aqueous Weathering Measurements

#### 2.2.1 Field Sampling Approaches

Pore water sampling with lysimeters and the centrifugation approach started in January 2023 and continued until August 2024. Suction cup lysimeters (SK20; Meter, USA) were installed in the center of each plot, at 40 cm depth, below the main root zone to monitor the influence of plant inputs on solute composition. The maximum suction applied by a lysimeter equals 0.1 MPa.

Measurements from the lysimeters began in 2021 and involved extracting pore water from lysimeters across all plots during each site visit. However, SWC levels significantly influenced pore water extraction success rates. When soils were not saturated, many lysimeters failed to yield sufficient sample volumes for analysis. The proportion of successful extractions fluctuated between sampling campaigns, depending on the SWC at each lysimeter location (see Figure 1 as an example).

To combat this issue, the 16 plots described in Section 2.1 were selected for planned sampling campaigns, during which soil pore waters were extracted from soils via lysimeters and soil centrifugation. During each sampling time point, 20 L of water from an on-site aquifer were applied to a 1 m^2^ area around each lysimeter using a back-mounted sprayer to emulate a 20 mm rainfall event. The Green–Ampt equation^45^ was used to estimate the time needed for the water to reach the lysimeter cup and soil block depth (Figure S3 and equations S1 and S2). The initial soil water deficit was set at 0.3, while saturated hydraulic conductivity was prescribed at 5 mm h^–1,^ and the initial soil water potential was defined as 1000 mm. These parameter values were derived from soil textural and organic matter (SOM) observations (Section 2.1). SOM content was estimated from soil organic C using the van Bemmelen factor.^46^ After the predicted time interval, soil blocks were excavated to a 10 cm depth for centrifugation, and lysimeters were sampled by applying vacuum suction. This method enabled direct comparison between lysimeter and centrifugation extraction approaches, even in drier soil conditions that typically hindered sampling efforts.

Planned sampling was conducted in March 2023, October 2023, January 2024, and May 2024. Aquifer water samples used for irrigation were periodically collected and analyzed for their TA (*n* = 3) (Figure S4) and cation concentrations (*n* = 5) (Figure S5).

#### 2.2.2 Soil Centrifugation Protocol

During the planned lysimeter sampling campaigns, a single soil block (10 × 10 × 10 cm) was excavated from the 1 m^2^ area encompassing each lysimeter (*n* = 16 plots), after which lysimeters were sampled. Each block was wrapped in plastic to prevent water evaporation and stored at 4 °C.

In the laboratory, we homogenized soil from within each plot,^47^ filled 50 mL Falcon tubes with the soil (*n* = 6) and weighed them. These aliquots were then centrifuged at 6000 rpm with a 7.125 cm rotation axis length (z300 K; Hermle LaborTechnik GmbH) for 20 min^31^ to extract pore water.^37^ A time limit of 20 min was chosen, as after this point, water could not be sufficiently extracted from the field-saturated soils (Figure S6). Syringe filters with a 13 mm diameter and 0.45 μm pore size (Whatman Uniflo 13; Cytiva Ltd.) were used to remove particulate organic matter from the resultant supernatant.

#### 2.2.3 Analysis of Pore Waters

Centrifugation- and lysimeter-derived pore water samples underwent identical chemical analyses. Pore water TA was immediately determined following sample preparation via acidimetric titration.^22^ The pH probe (ISE HI 422, Hanna Instruments Ltd.) was calibrated for every 25 samples to ensure measurement precision and accuracy. HCO_3_^–^ concentrations were derived from pore water TA values.^22^ Base cation concentrations were quantified via ICP-OES (Avio 500, PerkinElmer Inc.), using samples preserved in 1% HNO_3_. For ICP-OES, measurements were within 0.01% of known concentrations. The accuracy and precision of analyses were verified using multielement standards.

### 2.3 Assessing Potential Centrifugation-Induced Perturbations to Pore Water Alkalinity

The centrifugation method of pore water extraction differs from the lysimeter method because additional mechanical force is applied to the whole soil sample, which could perturb pore water chemistry. There are two mechanisms by which centrifugation could alter the chemistry of soil pore waters in an ERW context. First, there is mechanical disruption of soil pore structure through the compression and collapse of pore networks, which release chemical constituents from mesopores and micropores which would not typically be extracted with lysimeters. Second, there is mechanical dissolution of feedstock grains that are held in the soil matrix. We assume that centrifuging soil samples that do not contain feedstock can generate mechanical soil disruption only, while centrifuging samples with feedstock results in both mechanisms. Therefore, two extraction coefficients (*C*_e_) were derived to quantify the magnitude of perturbation due to centrifugation, as described below.

#### 2.3.1 Determining an Extraction Coefficient to Account for the Effects of Centrifugation on Pore Water Chemistry

To assess the effects of centrifugation on soil pore water chemistry, we performed an experiment where soil subsamples were fully saturated in 50 mL centrifuge tubes (*n* = 6) with known quantities of ultrapure water, which had negligible alkalinity (Figure S4). Half of the samples were centrifuged at 6000 rpm for 20 min, using a centrifuge with a 7.125 cm rotation axis length (z300 K; Hermle LaborTechnik GmbH).^31^ This time frame assumes that sufficient mixing of pore waters from different pore size fractions occurs during centrifugation, enabling the extraction of composite samples that are comparable across feedstock application treatments. The physical redistribution and mixing process during centrifugation occurs rapidly relative to the time scale of mineral dissolution and ion exchange reactions, ensuring that the extracted pore water reflects the chemical composition established through slower weathering processes, rather than transient disequilibrium conditions induced by the extraction method itself. The negligible TA increase following the centrifugation of feedstock in ultrapure water (Figure S7) also confirms that the 20 min extraction period captures existing pore water TA, such that the centrifugation method is able to detect mineral weathering that occurred under field conditions. The remaining samples were set aside and remained stationary for 20 min to represent a control group. Note that the purpose of adding ultrapure water to all soils is to ensure that water can be recovered from the stationary control group, which otherwise would not provide sufficient volume for analysis. Both groups of solutions were then syringe filtered (13 mm diameter and 0.45 μm pore size; Whatman Uniflo 13; Cytiva Ltd.) and sampled for TA via acidimetric titration.^22^ Figure 3 displays a schematic of this experiment.

**Figure 3:**
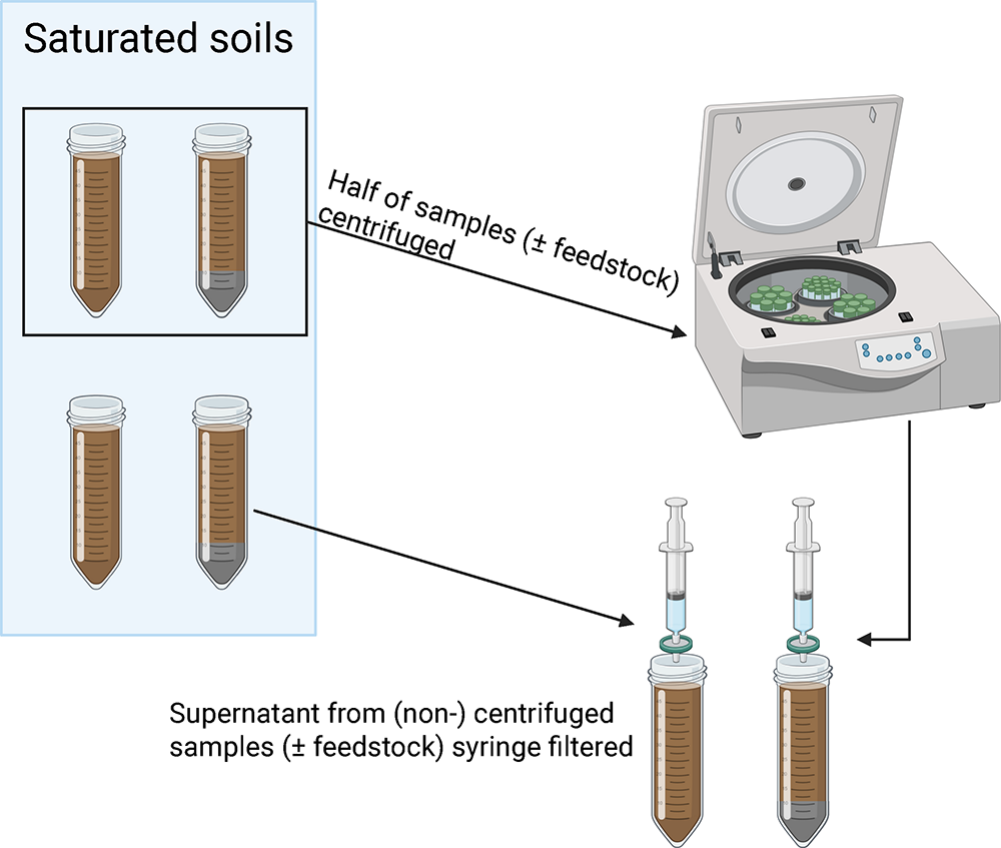
A pictographic description of the method used to obtain soil pore water and determine coefficient-transformed total alkalinity values resulting from centrifugation.

From this experiment, we derived an extraction coefficient for mechanical disturbance (*C*_e_) as the ratio of TA in the centrifuged versus non-centrifuged samples, as follows:

1 $$C_{e}=\frac{{\mathrm{TA}}_{c}\cdot V_{\mathrm{soil},\mathrm{nc}}\cdot \left(V_{\mathrm{soil},c}+V_{\mathrm{add},c}\right)}{{\mathrm{TA}}_{\mathrm{nc}}\cdot V_{\mathrm{soil},c}\cdot \left(V_{\mathrm{soil},\mathrm{nc}}\cdot V_{\mathrm{add},\mathrm{nc}}\right)}$$

Where TA (m_eq_ L^–1^) corresponds to the total alkalinity of the specific centrifugation treatment (nc = not centrifuged, c = centrifuged). *V* corresponds to the initial soil water volume (L). *V*_add_ relates to the volume of added ultrapure water that enables the soil samples to reach saturation. However, this term is only necessary when soils are saturated in the laboratory, to provide comparisons between centrifugation and lysimeter pore waters. Detailed equation derivations can be found in Supplementary Section 6.

C_e_ was calculated separately for samples from plots with (*C*_e,mech,diss_) and without (*C*_e,mech_) feedstock , under the assumption that a difference in the magnitude of *C*_e_ between sample types would reflect weathering-induced alterations to meso- and micropore water chemistry. *C*_e,mech_ is used to quantify mechanical disruption of soil only, while *C*_e,mech,diss_ is used to quantify the chemical impacts of the mechanical disruption of soils containing weathered feedstock. Despite the large variability in gravimetric soil moisture (GSM) among samples used to derive extraction coefficients (Figure S9), the variability among *C*_e,mech_ values remain small (0.89 ± 0.11). Notably, *C*_e,mech_ is approximately an order of magnitude less than C_e,mech,diss_ (9.77 ± 0.68), demonstrating that mechanical effects of centrifugation are negligible, and highlighting the fact that feedstock alters the chemistry of water in meso- or micropores, which can only be extracted under high tension.

The magnitude of extraction coefficients was unrelated to soil moisture content (*p* > 0.05) (Figure S8), indicating that variation in *C*_e_ is not systematically related to soil saturation. Therefore, we applied a mean extraction coefficient to field-derived TA data to account for the effects of centrifugation and enable comparisons with lysimeter-extracted pore water TA, using the following equation:

2 $${\mathrm{TA}}_{\mathrm{corr}}=\frac{\mathrm{TA}}{\overset{®}{C_{e}\;}}$$

where TA_corr_ (m_eq_ L^–1^) represents transformed TA values, calculated by dividing original TA values (TA; m_eq_ L^–1^) by the mean extraction coefficients ( $\overset{®}{C_{e,\mathrm{mech}}\;}$ or $\overset{®}{C_{e,\mathrm{mech},\mathrm{diss}}\;}$ ) derived from four samples (*n* = 4 for each coefficient).

For soil samples collected in plots without feedstock, $\overset{®}{C_{e,\mathrm{mech}}\;}$ converges to approximately one, indicating negligible effects of centrifugation on soil disaggregation and its impact on pore water TA. Therefore, we did not apply the extraction coefficient, $\overset{®}{C_{e,\mathrm{mech}}\;}$ , to samples from these plots prior to comparison with lysimeter-derived data. This convergence also indicates that TA is relatively uniformly distributed across macro- and micropores in soils that have not been amended with feedstock. However, $\overset{®}{C_{e,\mathrm{mech},\mathrm{diss}}\;}$ is approximately an order of magnitude higher, demonstrating that centrifugation extracts pore water with higher TA than gravimetrically drained macropore water (considered analogous to lysimeter-derived pore water, based on the tension it exerts). Since centrifugation applies a higher tension, this disparity suggests that feedstock-derived alkalinity is preferentially concentrated in micropores.

#### 2.3.2 Accounting for the Mechanical Disturbance of Feedstock via Centrifugation

The centrifugation method could artificially elevate the alkalinity of soil pore waters in samples from feedstock-amended plots, thereby inflating the treatment signal, if undissolved feedstock grains in the soil are mechanically weathered by centrifugation. To control for this, we performed a sub-experiment to quantify alkalinity perturbation due to the mechanical dissolution of feedstock during the 20 min extraction process. For this experiment, 50 mL of ultrapure water was decanted into 50 mL centrifuge tubes (*n* = 6), followed by increasing rates of feedstock addition (0.008, 0.022, 0.052, 0.066, and 0.080 g mL^–1^, with the uppermost value equivalent to 40 t ha^–1^ field application rate) (Figure S7). The solution was agitated gently by inversion and allowed to settle for approximately 24 h. A 5 mL supernatant subsample was then syringe filtered (13 mm diameter and 0.45 μm pore size; Whatman Uniflo 13; Cytiva Ltd.) for TA analysis. The remaining sample was centrifuged at 6000 rpm for 20 min, using the procedure described in Section 2.2.2 and sampled again for TA via acidimetric titration.^22^

We regressed the difference in TA between centrifuged and non-centrifuged samples against the concentration of feedstock in each pair, finding a significant positive relationship (*R* ^2^ = 0.72, *p* < 0.05). The slope of this regression indicates that centrifugation artificially increases measured TA values in feedstock-treated soils at a rate of 5.5 × 10^–4^ m_eq_ g^–1^ feedstock, at the centrifugation speed and timing used for our experiments. Assuming a maximum feedstock concentration of 0.08 g mL^–1^ at the 40 t ha^–1^ field application rate (which reflects the assumption that all feedstock remained in the top 10 cm of soil excavated for centrifugation), this would imply that mechanical weathering of feedstock grains in our sampled soils would contribute 0.04 m_eq_ L^–1^ to our TA measurements. This value is approximately an order of magnitude smaller than the measured TA values and falls within the measurement uncertainty for TA (Section 3.1).

### 2.4 Statistical Analyses

Simple linear regressions were used to evaluate the relationship between lysimeter-derived TA, the original centrifuge-derived TA (TA_c_) and coefficient-transformed TA (TA_corr,mech_). Separate one-tailed *t* tests were performed on centrifugation and lysimeter data sets to assess whether in situ feedstock application affects pore water TA and base cation concentrations. Base cation concentrations sampled via centrifugation were not coefficient-transformed, as *C*_e_ was only determined in terms of TA.

Statistical outliers were identified using Cook’s distance and Bonferroni outlier tests. Observations were removed from the analysis if a Bonferroni test *p*-value was <0.05 or a Cook’s distance value was >1. All parametric tests were checked to ensure that the data at least displayed homogeneity of variance using Levene’s test. If this assumption was violated, the data for the response variables were Box-Cox transformed. All statistical analyses used R version 4.1.1,^48^ using the Tidyverse^49^ and car^50^ packages.

## 3 Results and Discussion

### 3.1 Pore Water Extraction Method Comparison

Pore water TA varied by extraction method. In soils from feedstock-amended plots, lysimeter-extracted pore waters displayed TA values ranging from 0.0202 to 0.110 (mean: 0.076 ± 0.009) (Figure 4), while soils from non-feedstock-amended plots displayed TA values between 0.030 and 0.0898 (mean: 0.063 ± 0.006). By contrast, centrifugation-extracted pore waters from feedstock-amended soils exhibited a greater range of TA values, from 0.111 to 0.606 (mean: 0.343 ± 0.056). Similarly, non-feedstock-amended soils subject to centrifugation displayed pore water TA values ranging from 0.061 to 0.575 (mean: 0.210 ± 0.052).

**Figure 4:**
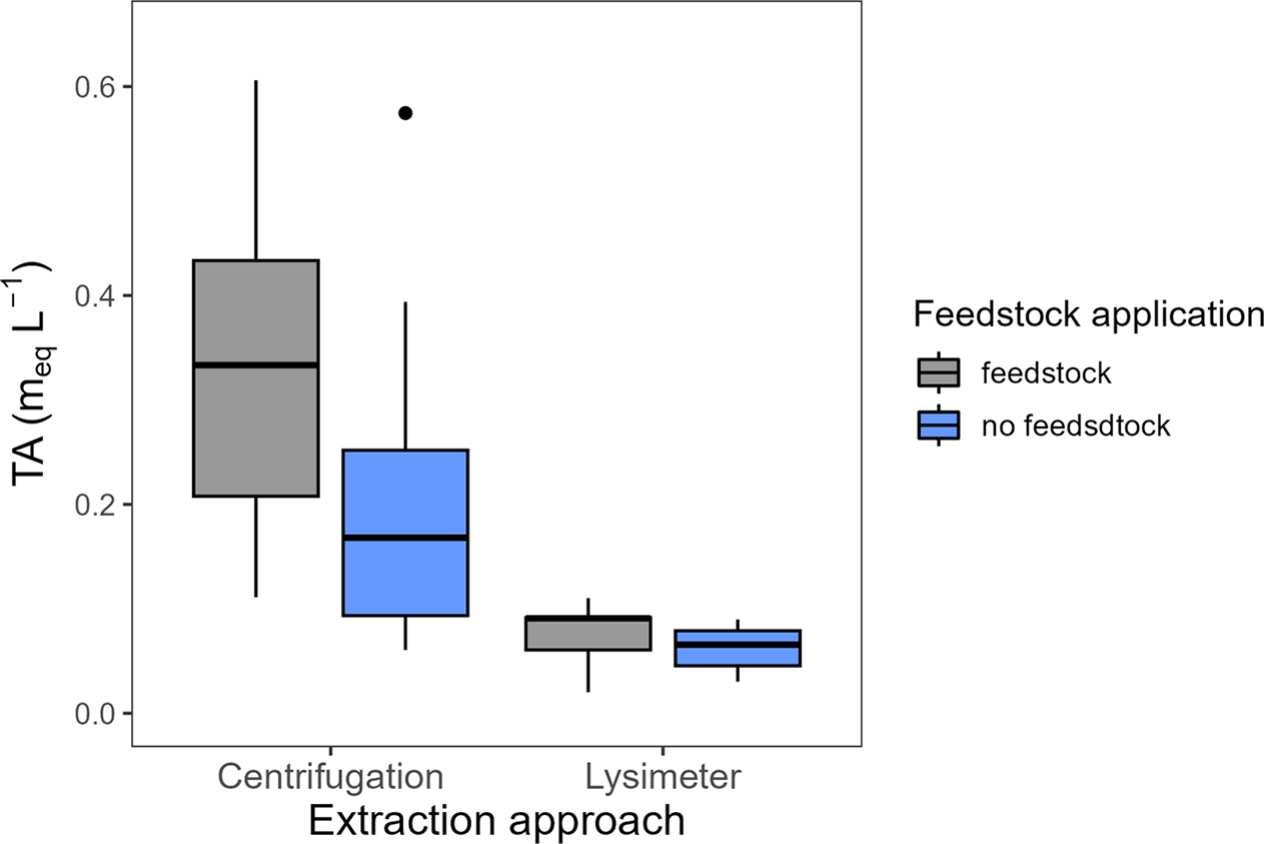
In situ soil pore water total alkalinity (TA) using centrifugation and lysimeter extraction methods from feedstock (gray) and no feedstock (blue) treatments.

Feedstock addition significantly elevated TA by 39% in pore waters sampled via centrifugation (*t* = 2.04, *p* = 0.03; Figure 5). However, no effect of feedstock addition was observed in the extraction coefficient-transformed TA (*t* = −3.37, *p* = 1; Figure 5). Similarly, lysimeters could not discern differences in TA between feedstock treatments (*t* = 1.15, *p* = 0.13; Figure 5).

**Figure 5:**
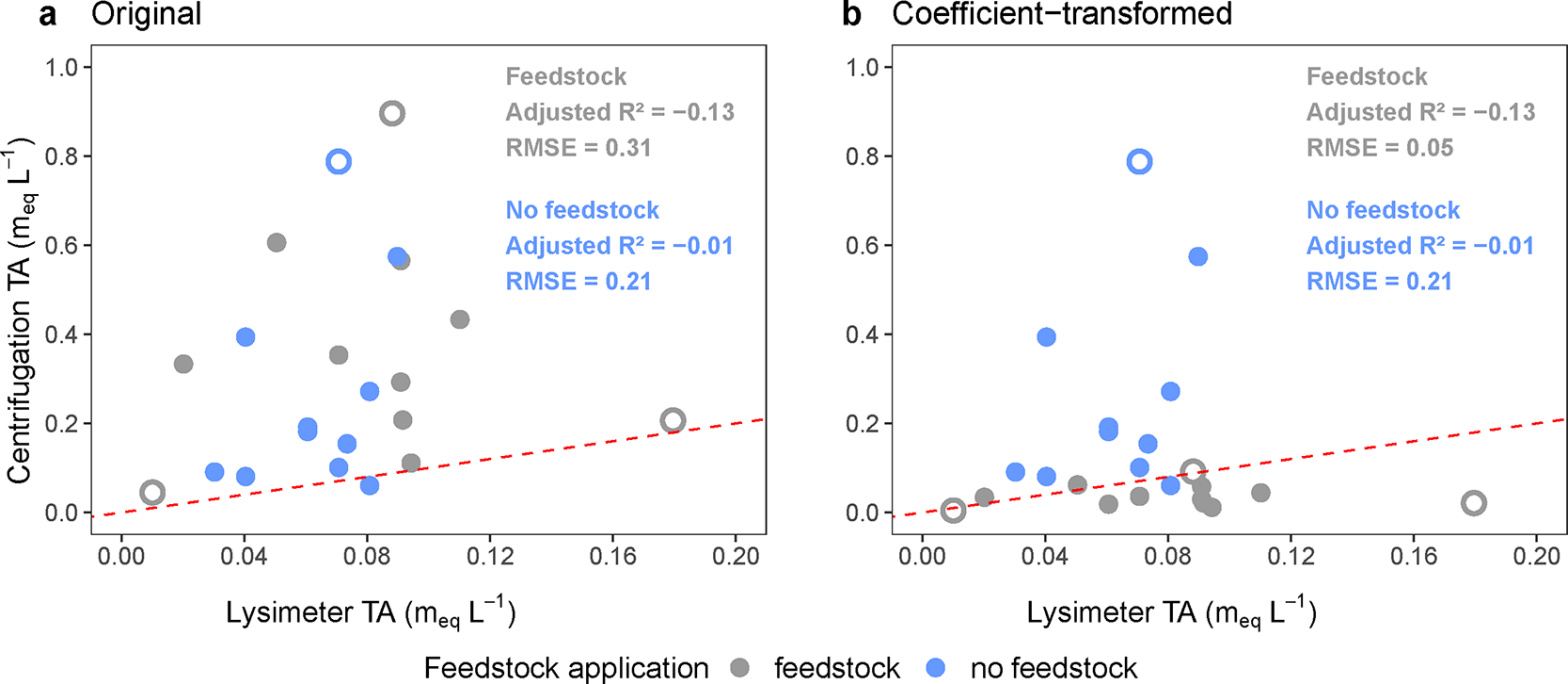
Relationship between lysimeter- (a) original and (b) extraction coefficient-transformed centrifugation-derived soil pore water total alkalinity (TA) from plots with (gray) and without (blue) feedstock applied. Adjusted *R* ^2^ is the adjusted coefficient of determination. RMSE is the root-mean-square error. The red dotted line represents a 1:1 association between the extraction approach TA. The hollow points represent statistical outliers.

Feedstock application increased Ca concentrations by 49% (*t* = 2.97, *p* < 0.01) in pore waters sampled via centrifugation. However, no effect was observed on other base cation concentrations (*p* > 0.05; Table S2). Feedstock application did not influence base cation concentrations in pore waters sampled via lysimeters (*p* > 0.05; Table S2).

Lysimeter and centrifugation TA values were unrelated in both feedstock-amended (*p* = 0.76) and non-amended soils (*p* = 0.36; Figure 5a), and this remained true after coefficient transformation (*p* = 0.76 and *p* = 0.36, respectively; Figure 5b). However, the coefficient-transformed TA values in feedstock-amended soils ranged from 0.011 to 0.062 (mean: 0.035 ± 0.006), whereas in non-amended soils, centrifugation-extracted values remained higher, ranging from 0.061 to 0.575 (mean: 0.210 ± 0.052). This suggests that while lysimeter and centrifugation methods yield systematically different values, coefficient-transformation can align their distributions in feedstock-treated soils, although individual sample variability remains evident.

Centrifugation pore water extraction provides a robust way to detect alkalinity release from feedstock dissolution under field conditions, revealing elevated pore water TA and Ca concentrations resulting from feedstock dissolution in an ecosystem-scale ERW experiment (Figure 4). The centrifugation method also demonstrated adaptability and potential to address current limitations in aqueous-phase sampling approaches.^49–51^ However, there was poor agreement between TA in pore waters sampled with centrifugation versus the traditional lysimeter-based method. Below, we first consider why the two methods may yield different estimates of pore water alkalinity. Then, we assess potential sources of error in pore water chemistry induced by centrifugation and explain why the method is likely robust to these errors. Finally, we discuss why the centrifugation approach may be a more sensitive and flexible option to assess weathering rates in situ.^52,53^

### 3.2 Differences in Pore Water Chemistry Measured with Lysimeters versus Soil Centrifugation

Pore water TA and base cation concentrations extracted via centrifugation were approximately 4 to 50 times higher than in water extracted with lysimeters. Moreover, the relationship between pore water TA measured via the two methods was poor, with non-significant relationships found in all cases (*p* > 0.05) (Figure 5). The two methods exert different soil water tensions and sample different soil pore size classes, which may contain water with different ion concentrations. Centrifugation at 6000 rpm, with a rotation axis length of 7.125 cm, applies a suction of 0.3 MPa to the soil, generating aforce that can extract water from pores as small as 0.1 μm in diameter.^31,37^ This is well below the micropore size threshold of 30 μm,^54^ indicating that centrifugation extracts alkalinity from both macro- and micropores. As water moves through the sample during parts of the centrifugation process, soil compaction occurs,^55^ further impacting pore size distribution and potentially liberating alkalinity from micropores.

Meanwhile, the maximum tension exerted by lysimeters is approximately 0.1 MPa, typically only sufficient to extract water from the largest pore size classes.^37^ Due to the high surface area-to-volume ratios of micropores, weathering products can remain trapped until the soil becomes sufficiently saturated for drainage to occur at low matric potentials.^56^ The extent of soil saturation depends on rainfall frequency and intensity,^57,58^ meaning weathering products may only be flushed from micropores during periods of heavy rainfall. Lysimeters can sample water exiting the vadose zone and estimate the export of weathering products from the soil column. However, these products may only be transported from micropores into macropores and detected by lysimeters during high-rainfall events. In contrast, by generating high capillary pressures, centrifugation provides an aqueous-phase estimate of the potential export of weathering products under hydroclimatic conditions that are not otherwise conducive to lysimeter sampling (refer to Figure 1 as an example).

The variation in pore water chemistry between extraction methodologies may also be attributable to different sampling depths associated with each method and corresponding variations in soil physicochemical and hydraulic properties.^10,59–61^ Soils for centrifugation were excavated to 10 cm depth, whereas lysimeters were installed from 30 to 40 cm depth. When we quantified TA variation with depth in 3 soil cores sampled from a non-feedstock amended plot, we found that TA was 85% higher in the top 10 cm of soil versus the 10–20 and 20–30 cm depth increments (Figure S10, *F*_2,6_ = 81.0, *p* < 0.001). This pattern probably reflects complex changes in decomposition, cation sorption, and secondary mineral formation with depth. Therefore, the higher TA values obtained via centrifugation likely reflect, in part, the shallower depths at which pore water was sampled with this method in feedstock and non-feedstock amended plots alike.

The variation in soil pore water chemistry with depth, if also present in feedstock-amended plots, could reflect the dilution of weathering products by higher SWC in deeper layers. To investigate this possibility, ERA5-Land reanalysis data^41^ were used to estimate SWC at depths corresponding to lysimeter and centrifugation sampling. ERA5-Land SWC is determined for discrete soil depths. As such, the centrifugation depth was represented as 0–7 cm, while the lysimeter depth was 7–28 cm. However, we did not find support for the hypothesis that SWC varied systematically through the profile. Nonetheless, as the feedstock was applied to the soil surface, a greater proportion of it may be located in upper soil layers, leading to considerably greater pore water TA upon dissolution in the field. Although weathering products migrate vertically through the soil profile, smaller amounts are typically observed within deeper soil layers. For example, following wollastonite application to soil columns with varying irrigation regimes, Khalidy et al.^62^ observed reduced leachate Ca^2+^ and Mg^2+^ concentrations with increasing soil depth. Te Pas et al.^63^ conducted a 64-day mesocosm experiment using grassland soil and observed that the majority of weathering products were retained within the soil matrix. Only a minimal fraction of weathering products entered the soil leachate, suggesting limited vertical transport. Similarly, Hasemer et al.^64^ found that soils from a pine forest and a clay loam exhibited comparable retention, with most weathering products remaining in situ. Under field conditions, Taylor et al.^6^ reported that HCO_3_^–^, Si, and base cations derived from silicate amendments in forest soils showed limited translocation beyond 30 cm depth. Together, these studies highlight an expanding line of empirical observations suggesting that a substantial fraction of ERW products is retained in the upper soil column, particularly on short- to midterm time scales. However, the number of long-term, field-scale investigations exploring the mobility of weathering solutes through soil profiles remains limited. Future work should aim to resolve the time scales over which solute export occurs and assess whether specific soil types or climatic regimes enhance downward fluxes of weathering products, as well as the vertical variation in leachate properties.^65,66^

Moreover, soil physicochemical properties that vary with depth, such as porosity, pore size distribution, soil texture, and organic matter content,^63,64^ may influence the export of weathering products from soil pores. For example, upper soil (O and A) horizons typically contain more organic matter and a larger proportion of macropores, in contrast to subsoils (B and C horizons), which often have lower porosity and hydraulic conductivity,^67,68^ reducing the rate of weathering product leaching from the soil column. This variation highlights the need to account for solute transport across the soil vertical profile to robustly estimate variation in leachate properties.

### 3.3 Pore Water Chemistry Measured via Soil Centrifugation is Robust to Mechanical Perturbation

To use centrifugation as a reliable method for soil pore water extraction, it must be ensured that centrifugation does not perturb soil pore water chemistry in ways that might affect subsequent analyses. Such perturbations could occur if: (1) the rotational force applied further dissolves rock grains, artificially augmenting the enhanced weathering signal, or (2) the process of centrifugation has mechanical effects on soil structure, resulting in the release of sorbed compounds from clays and soil organic matter surfaces.^69^ We demonstrate that neither of these scenarios occurred.

As detailed in Section 2.3.2, centrifugation of pure feedstock grains slightly elevated pore water TA. Given the observed release of alkalinity (standardized to feedstock concentration) and a feedstock application rate of 40 t ha^–1^ at our study site, the maximum TA generated by mechanical weathering could be calculated. However, this value is approximately an order of magnitude lower than the observed difference in TA from plots with and without feedstock (Figures 4, 5, and S7). We can, therefore, conclude that the “signal” from in situ ERW is very robust to the extremely small perturbations in pore water chemistry that occur due to centrifuge-induced feedstock weathering.^70^

We further demonstrated that soil centrifugation does not perturb pore water TA through changes to the physical structure of soil undergoing centrifugation (Figure S8). Mean differences in the TA of pore water from paired centrifuged and non-centrifuged samples were extremely small when focusing on the subset of soils which had not received feedstock in situ (Figure S8). By contrast, pore water TA values varied on average 10-fold between centrifuged and non-centrifuged samples collected from plots where feedstock was applied. This is consistent with our conclusion above: the products of feedstock dissolution accumulate in soil micropores, which can only be released by the high soil water tensions applied via centrifugation (but not with lysimeters). The difference between $\overset{®}{C_{e,\mathrm{mech}}\;}$ and $\overset{®}{C_{e,\mathrm{mech},\mathrm{diss}}\;}$ values (Figure S8) indicate that feedstock weathering produces spatially heterogeneous weathering products across the soil pore size distribution, with higher concentrations retained in smaller pores that require greater extraction pressures. This pattern highlights that micropores provide optimal conditions for dissolution reactions due to prolonged water residence times and increased mineral–water contact.^71^ However, the tendency of micropore waters to reach near-saturation may reduce dissolution rates over time as weathering products accumulate. In contrast, macropores support rapid water movement^72^ but limited mineral-water interactions. The preferential buildup of weathering products in micropores suggests they serve as primary reaction sites or storage reservoirs, while macropores function mainly as transport pathways.

For soils not amended with feedstock, centrifugation has only minor impacts on pore water chemistry (Figure S8). The mechanical dissolution of silicate rock and centrifuge-induced perturbation of soil structure will vary depending on the characteristics of the feedstock and the soil being studied. Therefore, we suggest that researchers using the centrifugation method follow the calibration protocol outlined in Section 2.3.

Future work could investigate the application of extraction coefficients to centrifugation-derived values across a wider range of soil types, feedstock, hydroclimatic conditions, and centrifugation parameters to ensure robustness and applicability. For example, the effects of feedstock dissolution rates on TA are influenced by mineralogy and particle size, with finer particles weathering faster,^10^ potentially impacting the extent to which centrifugation augments the enhanced weathering signal. Furthermore, soils with different physicochemical properties, such as texture and water content, may respond differently to the mechanical forces exerted during centrifugation, potentially yielding extraction coefficient values that differ from those observed in this study. This suggests the need to tune extraction coefficients based on site-specific conditions. As weathering rates are highly climate-dependent, primarily driven by precipitation and temperature,^8^ regional adjustments may also be necessary for MRV protocols.

### 3.4 Comparative Advantages of the Soil Centrifugation Approach

Seasonal variations in soil water content can preclude water sampling with low-tension lysimeters, limiting the ability to monitor continuous weathering fluxes. Temporal gaps in data often obscure the relationship between hydroclimatic conditions and weathering rates.^73^ By applying high tension to pore water, centrifugation successfully extracted sufficient sample volume across various soil moisture conditions. This ensured data collection across a wider temporal range of environmental conditions, which is critical for upscaling ERW MRV protocols.^20,28^

The greater sensitivity of centrifugation to TA, displayed by its ability to detect increased TA in plots with and without feedstock amendment, suggests that it provides more detailed insight into weathering-derived solutes in pore water (Figure 4).^74^ The elevated TA and Ca concentrations following centrifugation of feedstock-treated soils suggest an increase in HCO_3_^–^ concentrations. Therefore, when compared to lysimeters, centrifugation may be more effective at detecting the influence of feedstock amendment on pore water TA. This enhances its applicability across different deployment scenarios and highlights its suitability for use within the MRV sector.

A primary objective was to develop a protocol that can effectively analyze pore water chemistry under any soil water condition, and the observed increases in pore water TA (Figure 4) and Ca concentrations (Table S2) provide valuable insights about soil biogeochemistry regardless of the specific mineral source of these ions. Characterization of the feedstock (Supplementary Section 1.2) indicates that elevated Ca in pore waters from feedstock-amended plots may result from combined calcite and silicate mineral dissolution. Whatever the source of the Ca (calcite or silicates), centrifugation represents a robust detection method that is more sensitive to changes in pore water chemistry than lysimeters. Nonetheless, future studies would benefit from analyzing both base cation and anion concentrations, along with detailed feedstock characterizations, to better constrain the impact of centrifugation and lysimeters on pore water TA through alterations to aqueous-carbonate equilibria.

The cost of implementing centrifugation for MRV must be weighed against the benefits of improved alkalinity export process representation. Recent assessments indicate that protocols generating high-resolution data are more likely to be used to validate carbon-credit claims,^9,75^ offsetting greater initial costs. Centrifugation offers an accurate and low-cost method to estimate CDR, enhancing its applicability to scalable MRV protocols. Although the centrifugation method is inherently destructive and involves sampling at discrete depths, it can be incorporated into existing soil sampling practices, utilizing existing workflows and infrastructure such as those required to sample soil nutrients and pH. In turn, this sampling compatibility reduces operational costs associated with implementing a novel sampling methodology. Ultimately, to account for the drawbacks of aqueous- and solid-phase approaches,^18^ it is likely that a combination of both approaches will be needed to quantify CDR via ERW on a site-by-site basis robustly. Linking aqueous- and solid-phase analyses could connect estimates of weathering rates and product export dynamics, therefore enhancing the robustness of ERW estimates.^26^

## Supplementary Material



## Data Availability

The data and code related to this study are openly available in Zenodo at and http://doi.org/10.5281/zenodo.17722728.

## Notes

Additional experimental details, materials, and methods (PDF)
