# Supplementary material for: Utilizing Soil Centrifugation for Accurate Estimates of Carbon Dioxide Removal via Enhanced Rock Weathering
Source: Environ Sci Technol. 2025 Dec 15;59(50):27305–15. doi: 10.1021/acs.est.5c03699 (PMC12750519; doi:10.1021/acs.est.5c03699)
Supplement: Supplementary file 1 [file es5c03699_si_001.pdf]

Utilizing soil centrifugation for accurate estimates of carbon dioxide removal via enhanced rock weathering

Gregory Jones, Ziyan Zhang, Katherine Clayton, Lena Lancastle, Athanasios Paschalis, Bonnie Waring

Contents

Utilizing soil centrifugation for accurate estimates of carbon dioxide removal via enhanced rock weathering 1
1. Feedstock characterisation 1
1.1 Qualitative description 1
1.2 Semi-qualitative description via X-ray diffraction 2
1.3 Particle size distribution 3
2. Estimation of lysimeter sampling time after in situ irrigation 4
3. Irrigation water chemistry 5
4. Determination of centrifugation time interval 6
5. Mechanical feedstock dissolution and its total alkalinity release 7
6. Extraction coefficient derivation 8
6.1 Extraction coefficient for samples without the metabasalt feedstock 8
6.2 Extraction coefficient for samples with the metabasalt feedstock 9
7. Extraction coefficient comparison 10
8. Depth effects on pore water total alkalinity 11
9. Centrifugation pore water cation concentrations 11
References 12

1. Feedstock characterisation

1.1 Semi-qualitative description

The feedstock used in the present study is sourced from the local Builth Wells Quarry, developed in the Builth Volcanic Inlier; specifically from the Middle-Upper Ordovician Llanwedd Formation, of the Llanvirn-Caradoc stage. The quarry exposes three units: the Upper Basic Lava, a pyroclastic flow interpreted as an ignimbrite or lahar, and Lower Basic Lava units, equivalent to the Upper Spillite, Felsite Agglomerate, and Lower Spillite [1].

The rock is described as light-coloured, fine-grained to nearly aphyric, amygdaloidal, basaltic to andesitic lavas, chiefly composed of albite, chlorite, titanite, iron ore, and some clinopyroxene [2]. Amygdales are filled with secondary chlorite, calcite, quartz, rare albite and pumpellyite. Large phenocrysts of plagioclase dominate up to 50% of the rock locally, and have inclusions of white mica, calcite, clinozoisite, prehnite, titanite, pale green and brown chlorite, plus kaolinite [2,3]. The formation has been described as metamorphosed, invoking deuteric alteration of

hyaloclastites, metasomatism, and low-grade regional metamorphism, replacing primary minerals with weather-resistant secondary minerals [2–6]. The plagioclase is described as albite-oligoclase but is converted to pure albite in strongly metasomatised samples, and primary pyroxene and amphiboles are pseudomorphed by clusters of chlorite, titanite, and calcite[3]. It is also noted that biotite is altered to chlorite and titanite at the mineral edges [2].

1.2 Semi-qualitative description via X-ray diffraction

Semi-quantitative X-ray diffraction analyses reveal major quantities of plagioclase feldspar (58-87%) (Table S1). Sodium-endmember albite was most consistently matched with our feedstock diffractograms, but calcium-bearing intermediate labradorite and calcium-endmember anorthite also were a strong match, suggesting calcium plagioclase is also present, consistent with Nicholls [3]. The feedstock is clay-rich with 11-17% chlorite and trace amounts of vermiculite (1-2%). Trace amounts of quartz (1-5%) and titanite (2-5%) are also present, and variable amounts of calcite (1-14%).

Conditions used to determine feedstock mineralogy via XRD consisted of CuKα radiation, ran at 40 kV and 15 mA. These are continuous scans with 2θ ranging from 2.00° to 80.00°, using a step size of 0.01 ° and a scan speed of 0.0833°/s, performed with a 1° divergence slit. Semi-quantitative results are calculated using HighScore Panalytical software, which employs a referenced scale factor and Reference Intensity Ratio (RIR) values from the International Centre for Diffraction Data (ICDD) mineral database. This normalised RIR method [7] produces semi-quantitative results, estimating mass fractions using peak intensity values from feedstock ground to <90 microns (n = 5) [8]. Crystalline mineral phases were identified using PANalytical HighScore Plus software (v.4.8) linked to the International Centre for Diffraction Data PDF-5+ database [9].

Table S1: Mineralogical composition of feedstock applied to the site in November 2020, as determined by X-ray diffraction analysis.

Major >20% minor <20% trace <3%	Silicates										Carbonates/ Phosphates/ Sulfates/ Oxides
	Mafic minerals (Nesosilicates + Inosilicates)			Phyllosilicates		Tectosilicates					
				Mica	Clays	Feldsphathoids	Feldspars			Silica minerals	
							Alkali	Plagioclase			
	Olivine	Pyroxene	Amphibole	Biotite e.g.	Chlorite + Vermiculite	Nepheline, leucite e.g.	Orthoclase (K)	Albite (Na)	Anorthite (Ca)	Quartz	Calcite
Typical Basic Basalt	Minor	Major	Major-minor	minor-trace	not expected	minor-trace	minor-trace	Major-minor	Major	minor-trace	not expected
Llanelwedd Basalt					11-17%			58-87%		1-5%	1-14%

1.3 Particle size distribution

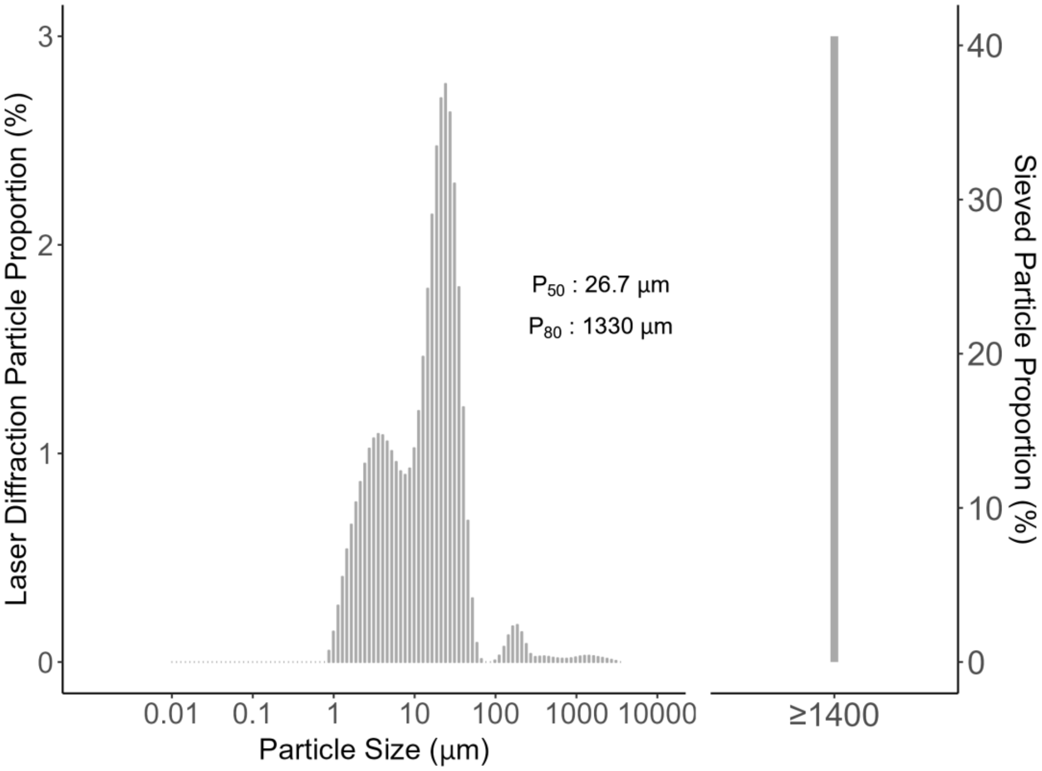

Figure S1: Particle size distribution of feedstock deployed in 2020, determined with a laser particle size diffraction analyser (<1.4 mm; n = 5) and sieving (>1.4 mm; n = 5). The p50 (median) and p80 values, respectively, represent the cumulative sum of the feedstock particle diameters in the lowest 50% and 80% ranges.

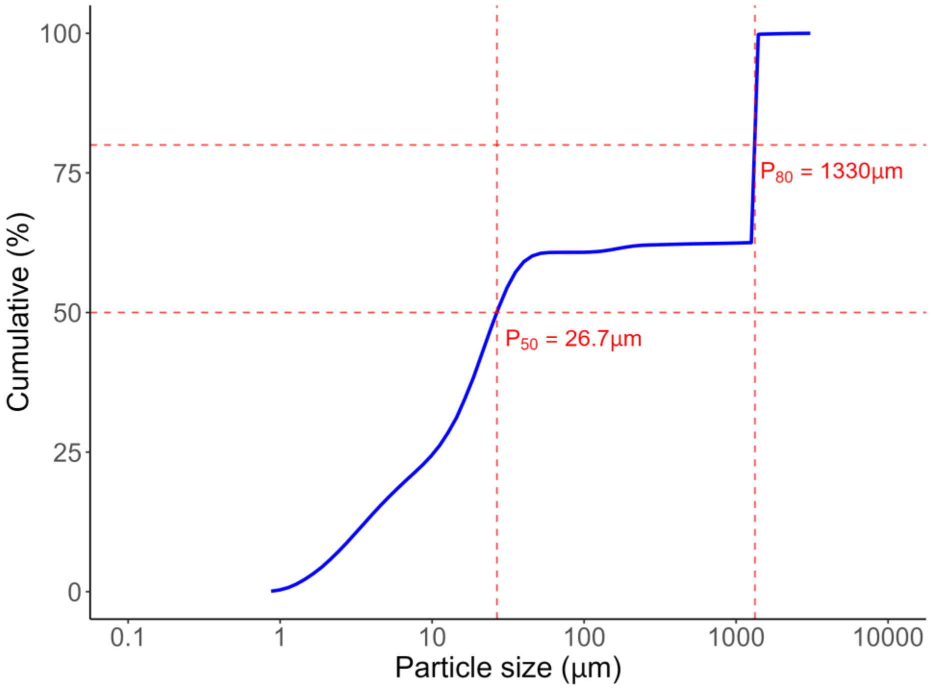

Figure S2: Cumulative particle size distribution for the feedstock deployed in 2020, displaying the p50 (median) and p80 values, which respectively represent the cumulative sum of the lowest 50% and 80% feedstock particle diameters.

2. Estimation of lysimeter sampling time after in situ irrigation

The Green-Ampt model was parameterised using values corresponding to the fine-textured loam soil present at the site [10]. The initial soil water deficit ($\Delta\theta$) was set at 0.3. Saturated hydraulic conductivity (K_s) was prescribed at 5 mm h^{-1}, while the initial soil water potential (Ψ) was defined as 1000 mm. Simulations ran for 5 hours, using a timestep (Δt) of 1 second. Cumulative infiltration (F) was iteratively calculated using an implicit form of the Green-Ampt equation [11]. Subsequently, the wetting front depth (L) was estimated from the cumulative infiltration and initial soil water deficit (Figure S3). Simulations were conducted in MATLAB version 24.1.0.2689473 (R2024a) Update 6 [12].

Green-Ampt equation:

$$F(t) = K_s t + \Psi \Delta\theta \ln\left(1 + \frac{F(t)}{\Psi \Delta\theta}\right) \qquad (S1)$$

Wetting front depth:

$$L(t) = \frac{F(t)}{\Delta\theta} \qquad (S2)$$

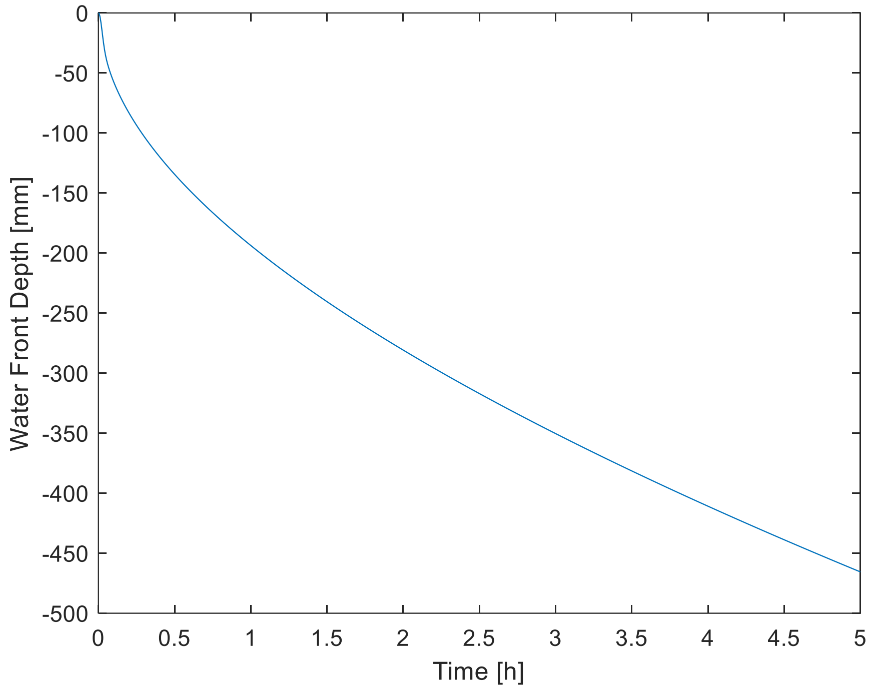

Figure S3. Time taken for the water front to reach a specific soil depth following the addition of 20 L of water, as calculated using the Green-Ampt equation.

3. Irrigation water chemistry

A one-tailed t-test was used to evaluate differences in TA between the irrigation water source and ultra-pure water. Water source A displayed 1400% higher TA than ultra-pure water ($t = 15.12$, $p < 0.01$).

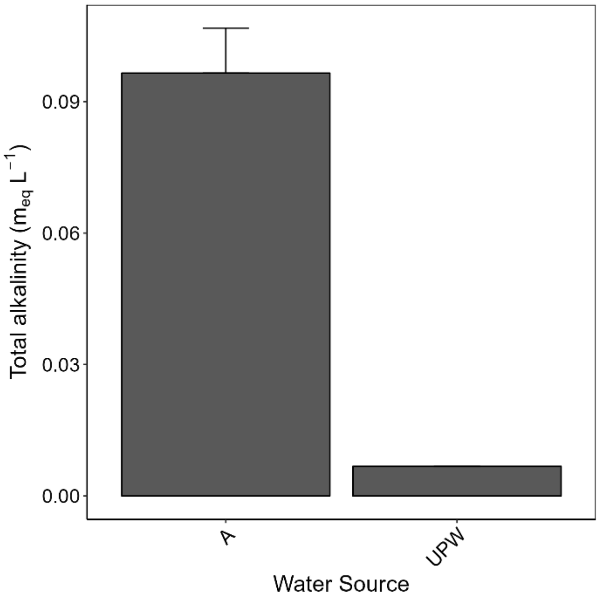

Figure S4: On-site aquifer water total alkalinity compared with ultrapure water (mean + standard error). Source A is a single well used to extract aquifer water. UPW denotes ultra-pure water, which was used as a control.

A two-way ANOVA was conducted to examine differences in cation concentrations between water source A and ultra-pure water (control) across sampling dates. Water source and sampling date were treated as additive factors. The results revealed significant differences among the water source A and ultra-pure water for Mg: $F(1,10) = 4.21$, $p = 0.07$, Ca: $F(1,12) = 5.67$, $p < 0.05$, Na: $F(1,12) = 6.18$, $p < 0.05$, K: $F(1,12) = 7.13$, $p < 0.05$, but not Sr: $F(1,10) = 28.9$, $p = 0.23$ (Figure S5). B within ultra-pure water is not displayed as concentrations were below the detection limits of ICP-OES.

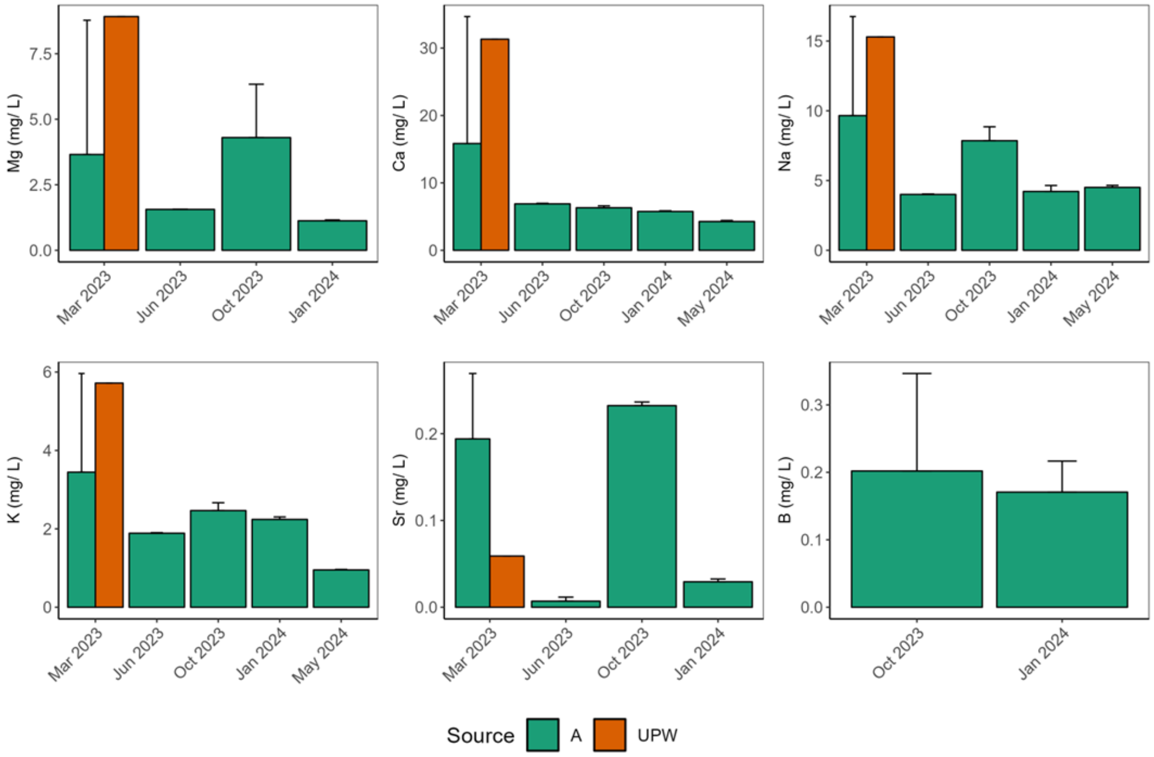

Figure S5: On-site aquifer water cation concentrations (mean + standard error) (a-f) across sampling dates. Source A is a single well used to extract aquifer water (green bars). UPW denotes ultra-pure water, which is used as a control (orange bar).

4. Determination of centrifugation time interval

Soil samples from October 2023 were subsampled for gravimetric soil moisture, which was determined at 65 °C [13]. Soil samples (n = 6) were then centrifuged using the method described in the primary methods Section 2. The supernatant was removed at two-minute intervals, and its volume was measured. The centrifuging continued at two-minute intervals until no further supernatant could be sufficiently extracted to perform additional analyses, such as acidimetric titration.

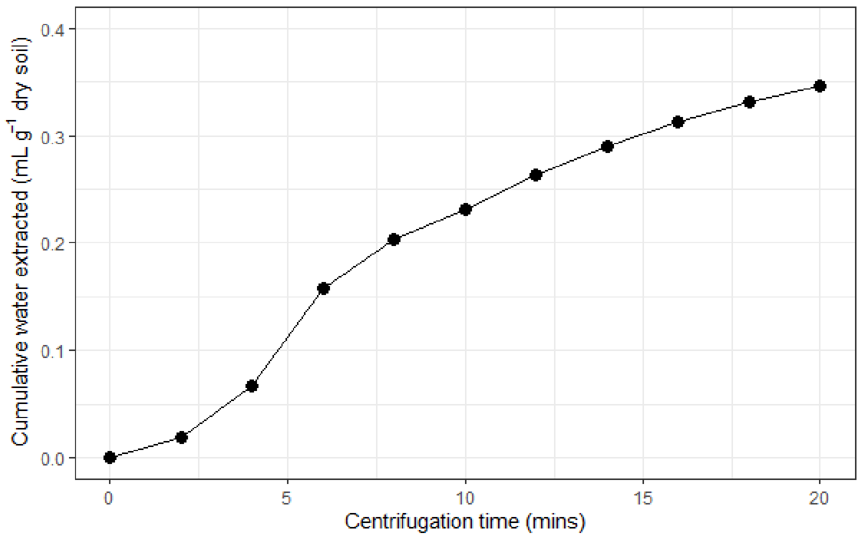

Figure S6: Cumulative pore water extracted (mL per g of dry soil) at two-minute timesteps via the centrifugation extraction method. Gravimetric soil moisture (GSM) = 29.6%.

5. Mechanical feedstock dissolution and its total alkalinity release

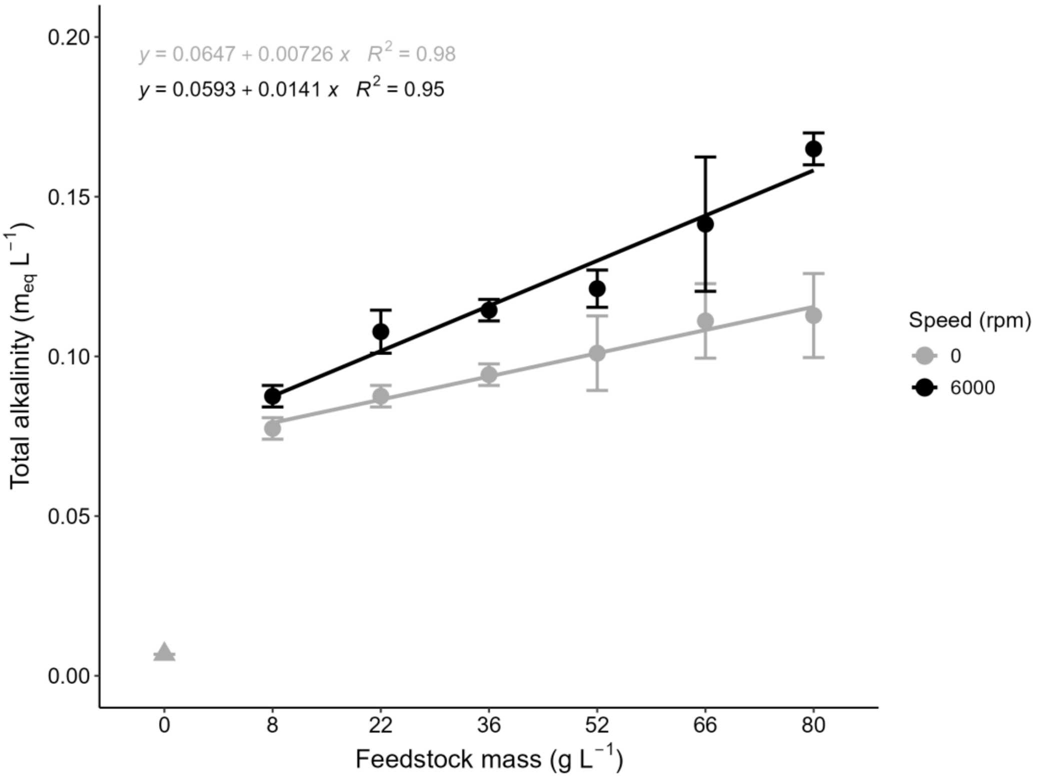

Figure S7: Total alkalinity (TA) measurements obtained by increasing masses of feedstock suspended in ultra-pure water and subjecting the mixture to the maximum centrifugation speed (6000 rpm; black points) versus a control group (0 rpm; grey points). For comparison, the TA of ultra-pure water alone is represented by the grey triangular point. Points show mean ± standard error.

6. Extraction coefficient derivation

6.1 Extraction coefficient for samples without the metabasalt feedstock

To calculate the impact of mechanical disruption of centrifugation on soil and its associated influence on pore water alkalinity ($C_{e,mech}$), two sets of soil samples without metabasalt were saturated with ultra-pure water. We derived extraction coefficients by accounting for the dilution effect of adding ultra-pure water to samples to reach saturation. This allowed for a greater yield of analysable supernatant following centrifugation.

The soils were divided into two subsamples: one was centrifuged, and the other was not, denoted by the subscripts *c* and *nc*. This yields two treatment groups (note that soils used in this experiment do not contain metabasalt, signified by the subscript *nb*), for example:

- $TA_{nc,nb}$ for the measured TA of samples with no centrifugation
- $TA_{c,nb}$ for the measured TA of samples with centrifugation

Here, we refer to $X_{c,nb}$ and $X_{nc,nb}$ as sets of measurements (*X*) from the same soil sample without metabasalt, but have either been centrifuged/ not centrifuged and have different initial soil water volumes.

The measured total alkalinity (TA_{nb}; m_{eq} L^{-1}) can be described as:

$$TA_{nc,nb} = \frac{M_{nc,nb}}{V_{nc,nb}} \qquad (S3)$$

$$TA_{c,nb} = \frac{M_{c,nb}}{V_{c,nb}} \qquad (S4)$$

Where the alkaline load (*M*) in the unit of m_{eq} is the product of total alkalinity in the unit of m_{eq} L^{-1}, TA and the total soil water volume (*V*) after saturation in the unit of L.

The alkaline load of centrifuged/ non-centrifuged soil samples following the addition of ultra-pure water alkaline load (M_{UPW}; m_{eq}) to the initial alkaline load of soil pore water (M_{soil}; m_{eq}) can be determined as:

$$M_{nc,nb} = M_{soil,nc,nb} + M_{UPW,nc,nb} \qquad (S5)$$

$$M_{c,nb} = M_{soil,c,nb} + M_{UPW,c,nb} \qquad (S6)$$

For centrifugation and non-centrifugation treatments, the alkaline load of ultra-pure water (M_{UPW}; m_{eq}) can be calculated as:

$$M_{UPW} = TA_{UPW} \,.\, V_{add} \qquad (S7)$$

Where, TA_{UPW} is the measured TA (m_{eq} L^{-1}) of ultra-pure water and V_{add} (L) the volume of ultra-pure water added for the sample to reach saturation. Therefore, the total alkalinity of soil pore water (TA_{soil}; m_{eq} L^{-1}) can be determined as:

$$TA_{soil,nc,nb} = \frac{TA_{nc,nb} \cdot (V_{soil,nc,nb} + V_{add,nc,nb}) - TA_{UPW} \cdot V_{add,nc,nb}}{V_{soil,nc,nb}} \quad \text{(S8)}$$

$$TA_{soil,c,nb} = \frac{TA_{c,nb} \cdot (V_{soil,c,nb} + V_{add,c,nb}) - TA_{UPW} \cdot V_{add,c,nb}}{V_{soil,c,nb}} \quad \text{(S9)}$$

In which and V_{soil} (L) denotes the initial soil water volumes of each sample. Consequently, the mechanical effects of centrifugation on soil disaggregation and its potential influence on pore water TA are estimated with the ratio of TA and soil water volumes within samples which have/ have not been centrifuged ($\frac{TA_{soil,c,nb}}{TA_{soil,nc,nb}}$; $C_{e,mech}$), derived from equations S8-9:

$$C_{e,mech} = \frac{TA_{c,nb} \cdot (V_{soil,c,nb} + V_{add,c,nb}) - TA_{UPW} \cdot V_{add,c,nb}}{TA_{nc,nb} \cdot (V_{soil,nc,nb} + V_{add,nc,nb}) - TA_{UPW} \cdot V_{add,nc,nb}} \cdot \frac{V_{soil,nc,nb}}{V_{soil,c,nb}} \quad \text{(S10)}$$

Since the TA of the ultra-pure water is negligible ($TA_{UPW} \cong 0$) compared to the soil pore water, and no water will be added to the centrifugation samples when applying the extraction coefficient, its minor influence ($- TA_{UPW} \cdot V_{add}$) is not significant enough to be represented in the final extraction coefficient equation.

$$C_{e,mech} = \frac{TA_{c,nb} \cdot V_{soil,nc,nb} \cdot (V_{soil,c,nb} + V_{add,c,nb})}{TA_{nc,nb} \cdot V_{soil,c,nb} \cdot (V_{soil,nc,nb} + V_{add,nc,nb})} \quad \text{(S11)}$$

6.2 Extraction coefficient for samples with the metabasalt feedstock

In our sub-experiment, soil samples containing metabasalt were saturated with ultra-pure water to calculate the combined mechanical impact of centrifugation on pore water alkalinity from soil disturbance and centrifuge-induced metabasalt dissolution ($C_{e,mech,diss}$). The soils were then divided into two subsamples: one was centrifuged, and the other was not, denoted by the respective subscripts *c* and *nc*. This yields two treatment groups (note that the soils used in this experiment contain metabasalt, as indicated by the subscript *b*). Consequently , the extraction coefficient for samples containing metabasalt ($C_{e,mech,diss}$) can be determined using the same process presented in Supplementary Section 5.1:

$$C_{e,mech,diss} = \frac{TA_{c,b} \cdot V_{soil,nc,b} \cdot (V_{soil,c,b} + V_{add,c,b})}{TA_{nc,b} \cdot V_{soil,c,b} \cdot (V_{soil,nc,b} + V_{add,nc,b})} \quad \text{(S12)}$$

7. Extraction coefficient comparison

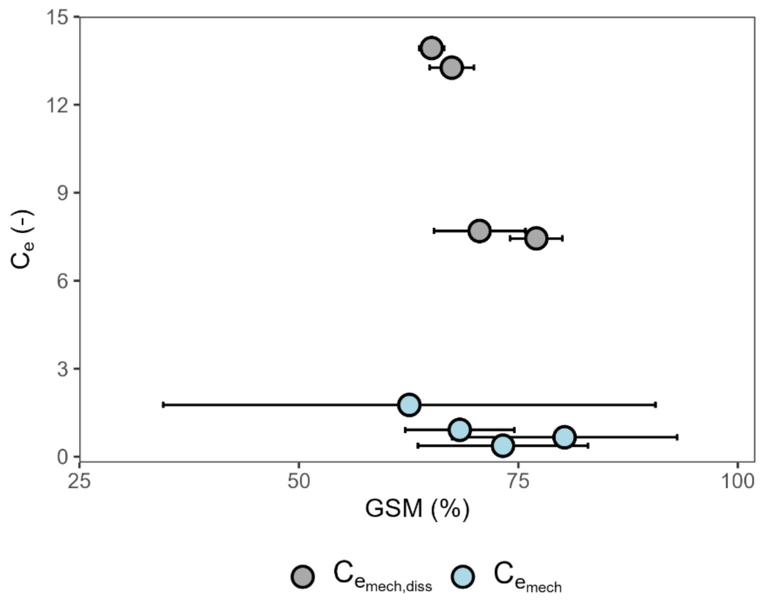

Figure S8: Comparison of extraction coefficient (Ce) values with respect to gravimetric soil moisture (GSM) (mean ± one standard deviation) across samples from metabasalt ($C_{e,mech,\ diss}$) / non-metabasalt ($C_{e,\mathit{mech}}$) treatments.

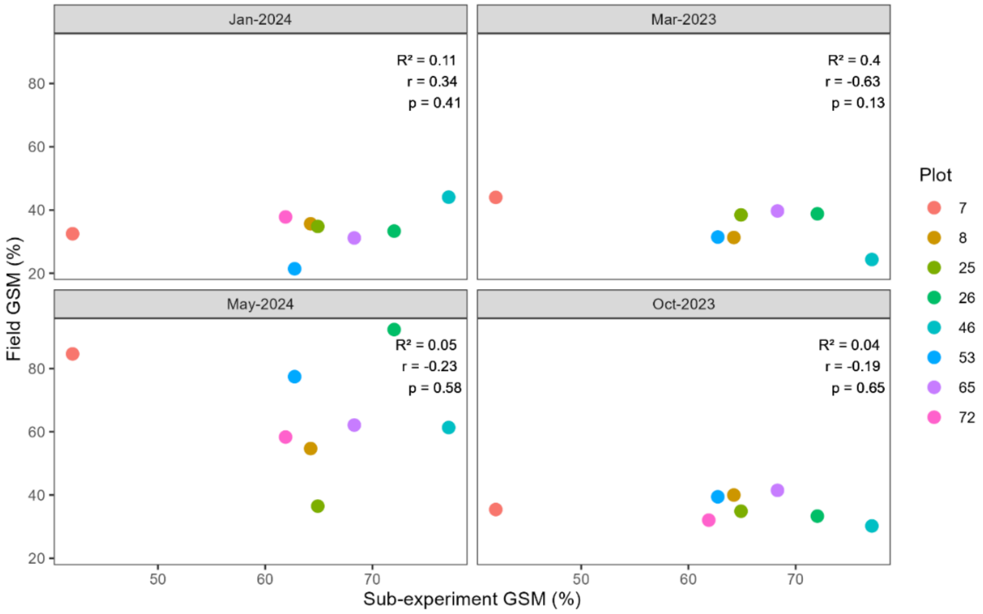

Figure S9: Association between gravimetric soil moisture (GSM) of samples used for the sub-experiment to determine extraction coefficient values and GSM values of field samples to which the extraction coefficients were applied, separated by sampling date. Different coloured points represent the sampled plots.

8. Depth effects on pore water total alkalinity

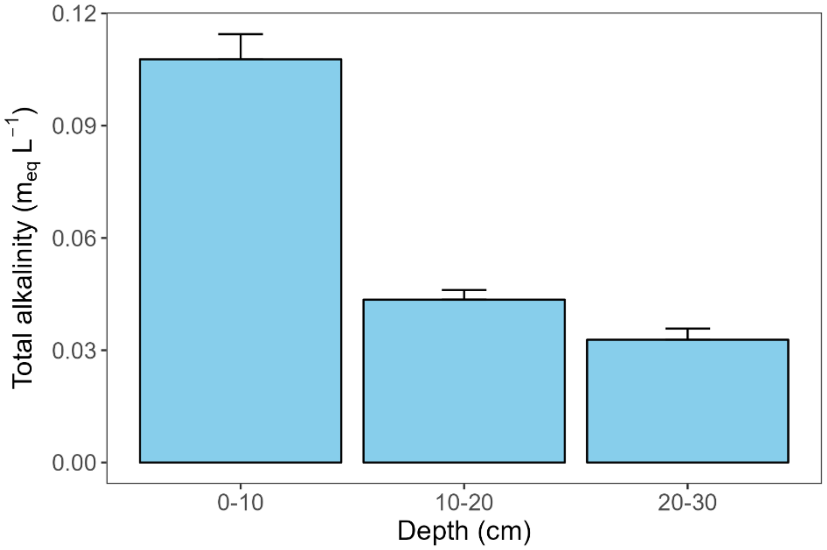

Figure S10: Pore water total alkalinity obtained via the centrifugation extraction approach, in samples collected at different soil depths from a non-metabasalt amended plot.

9. Centrifugation pore water cation concentrations

Table S2: Summary statistics and effect sizes for pore water cation concentrations in relation to in situ feedstock application, following one-tailed t-tests. Outliers were removed using the Bonferroni outlier and Cook's distance tests. Effect sizes are presented as the percentage change in mean pore water cation concentrations in the metabasalt treatment compared to the control (no metabasalt). Bold *p*-values correspond to significant treatment effects ($p < 0.05$).

Extraction approach	Cation	t-value	Degrees of freedom	*p*-value	Effect size (%)
Lysimeter	Mg	0.13	54	0.44	- 15.2
	Ca	0.80	48	0.21	+ 7.6
	Na	-0.89	66	0.81	+7.16
	K	-0.07	66	0.52	- 1.6
	Sr	-0.11	50	0.46	+ 11.7
	B	-1.43	26	0.92	+ 420.6
Centrifugation	Mg	-0.52	54	0.70	- 16.4
	Ca	2.97	57	**0.002**	+ 48.6
	Na	-1.45	55	0.92	- 18.6
	K	0.44	58	0.33	+ 10.3
	Sr	0.63	47	0.26	- 4.4
	B	-2.36	20	0.99	+ 28.2

References

(1) Jones, O. T.; Pugh, W. J. The Laccolithic Series. *American Journal of Science* **1949**, *247* (6), 353–371. https://doi.org/10.2475/ajs.247.6.353.

(2) Furnes, H. A Comparative Study of Caledonian Volcanics in Wales and West Norway. Doctoral Thesis, University of Oxford, Oxford, 1978.

(3) Nicholls, G. Autometasomatism in the Lower Spilites of the Builth Volcanic Series. *Quarterly Journal of the Geological Society of London* **1958**, *114* (1–4), 137–162. https://doi.org/10.1144/gsjgs.114.1.0137.

(4) Roberts, B. Low Grade and Very Low Grade Regional Metabasic Ordovician Rocks of Llŷn and Snowdonia, Gwynedd, North Wales. *Geol. Mag.* **1981**, *118* (2), 189–200. https://doi.org/10.1017/S0016756800034385.

(5) Bevins, R. E.; Kokelaar, B. P.; Dunkley, P. N. Petrology and Geochemistry of Lower to Middle Ordovician Igneous Rocks in Wales: A Volcanic Arc to Marginal Basin Transition. *Proceedings of the Geologists' Association* **1984**, *95.4*, 337–347.

(6) Bevins, R. E.; Rowbotham, G. Low-grade Metamorphism within the Welsh Sector of the Paratectonic Caledonides. *Geological Journal* **1983**, *18.2*, 141–167.

(7) Chung, F. H. Quantitative Interpretation of X-Ray Diffraction Patterns of Mixtures. I. Matrix-Flushing Method for Quantitative Multicomponent Analysis. *J Appl Cryst* **1974**, *7* (6), 519–525. https://doi.org/10.1107/S0021889874010375.

(8) Waring, B.; Averill, C.; Bidartondo, M.; Suz, L.; Beerling, D.; Crowther, T.; Lancastle, L.; Clayton, K.; Gobelius, L.; Jones, G.; Lindsay, O.; Steidinger, B.; Allen, H.; Nicholls, C. Microbiome Manipulation and Enhanced Weathering Stimulate CO2 Removal in Reforestation. Preprint at Research Square 2025. https://doi.org/10.21203/rs.3.rs-5982308/v1.

(9) International Centre for Diffraction Data. PDF-5+ - ICDD, 2024. https://www.icdd.com/pdf-5/ (accessed 2024-07-22).

(10) Saxton, K. E.; Rawls, W. J. Soil Water Characteristic Estimates by Texture and Organic Matter for Hydrologic Solutions. *Soil Science Society of America Journal* **2006**, *70* (5), 1569–1578. https://doi.org/10.2136/SSSAJ2005.0117.

(11) Green, W.; Ampt, G. A. Studies on Soil Physics. *J. Agric. Sci.* **1911**, *4* (1), 1–24. https://doi.org/10.1017/S0021859600001441.

(12) The Mathworks, Inc. MATLAB, 2024. https://uk.mathworks.com/products/matlab.html (accessed 2024-10-09).

(13) Black, C. A. *Methods of Soil Analysis: Physical and Mineralogical Properties, Including Statistics of Measurement and Sampling. Part 1*, 1st ed.; American Society of Agronomy, 1965.
